# Breast Conservation Therapy for Primary Squamous Cell Carcinoma of the Breast in an Elderly Woman: A Case Report

**DOI:** 10.7759/cureus.59447

**Published:** 2024-05-01

**Authors:** Bailey A Loving, Shaveena Sivapalan, Casey P Schukow, Jashan Khaira, Frank A Vicini, James Fontanesi

**Affiliations:** 1 Radiation Oncology, Corewell Health, Royal Oak, USA; 2 Radiation Oncology, Michigan State University, Auburn Hills, USA; 3 Pathology, Corewell Health William Beaumont University Hospital, Royal Oak, USA; 4 Radiation Oncology, Oakland University William Beaumont School of Medicine, Rochester Hills, USA; 5 Radiation Oncology, GenesisCare, Farmington Hills, USA; 6 Radiation Oncology, Corewell Health William Beaumont University Hospital, Royal Oak, USA

**Keywords:** personalized oncology treatment, immunohistochemistry in oncology, surgical margins in breast cancer, diagnostic imaging in breast cancer, elderly patient management, adjuvant hypofractionated radiotherapy, breast conservation therapy, triple negative breast cancer, primary squamous cell carcinoma, case report

## Abstract

This case report details a rare instance of primary squamous cell carcinoma (PSCC) of the breast in an octogenarian, emphasizing the unique diagnostic and treatment challenges posed by this malignancy in an elderly patient and adding to the scientific literature on PSCC managed with breast conservation therapy (BCT). An 80-year-old woman with medical comorbidities presented with a focal asymmetry in the right breast's retroareolar plane, detected during routine screening mammography. Diagnostic evaluations raised high suspicion for malignancy, confirmed as PSCC by ultrasound-guided biopsy. Histopathological analysis showed atypical keratinizing squamous epithelial nests and cysts. The patient underwent lumpectomy and re-excision of close surgical margins with a sentinel lymph node biopsy, which showed well-differentiated invasive squamous cell carcinoma with no residual carcinoma or nodal involvement. She was treated with adjuvant hypofractionated radiation therapy, experiencing minimal side effects. This case highlights the importance of considering individualized, nuanced approaches to adjuvant therapies in the treatment of PSCC in older patients. It demonstrates that BCT, coupled with carefully selected adjuvant therapy, can be a successful treatment strategy for PSCC in the elderly, contributing valuable insights into the management of this rare condition.

## Introduction

Primary breast squamous cell carcinoma (PSCC) is an extremely rare entity that accounts for less than 0.1% of all invasive breast cancers, with few cases reported in the current literature [1-4]. Known for its aggressive behavior, PSCC often presents with aggressive disease [5]. Despite its occurrence, the literature remains sparse, particularly regarding the management and outcomes of breast conservation therapy (BCT) for PSCC. In this report, we detail the case of an 80-year-old woman diagnosed with PSCC of the breast, aiming to enrich the sparse literature on BCT within this specific demographic.

## Case presentation

**Figure 1:**
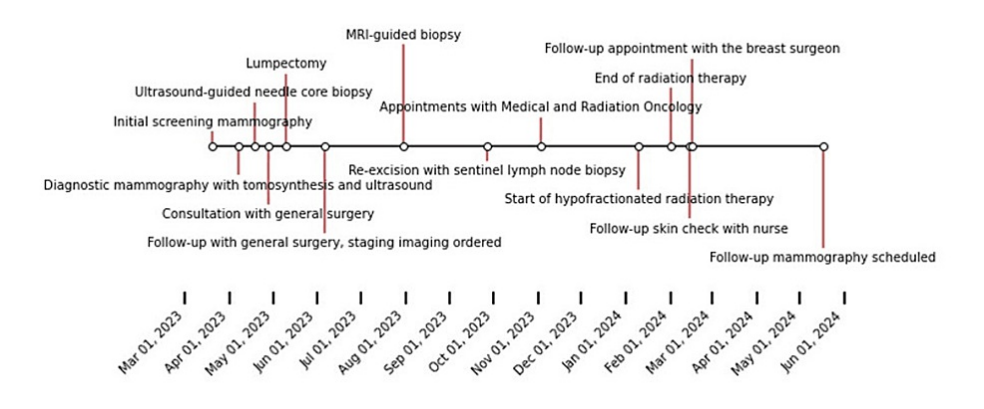
Timeline of patient’s care episode.

**Figure 2:**
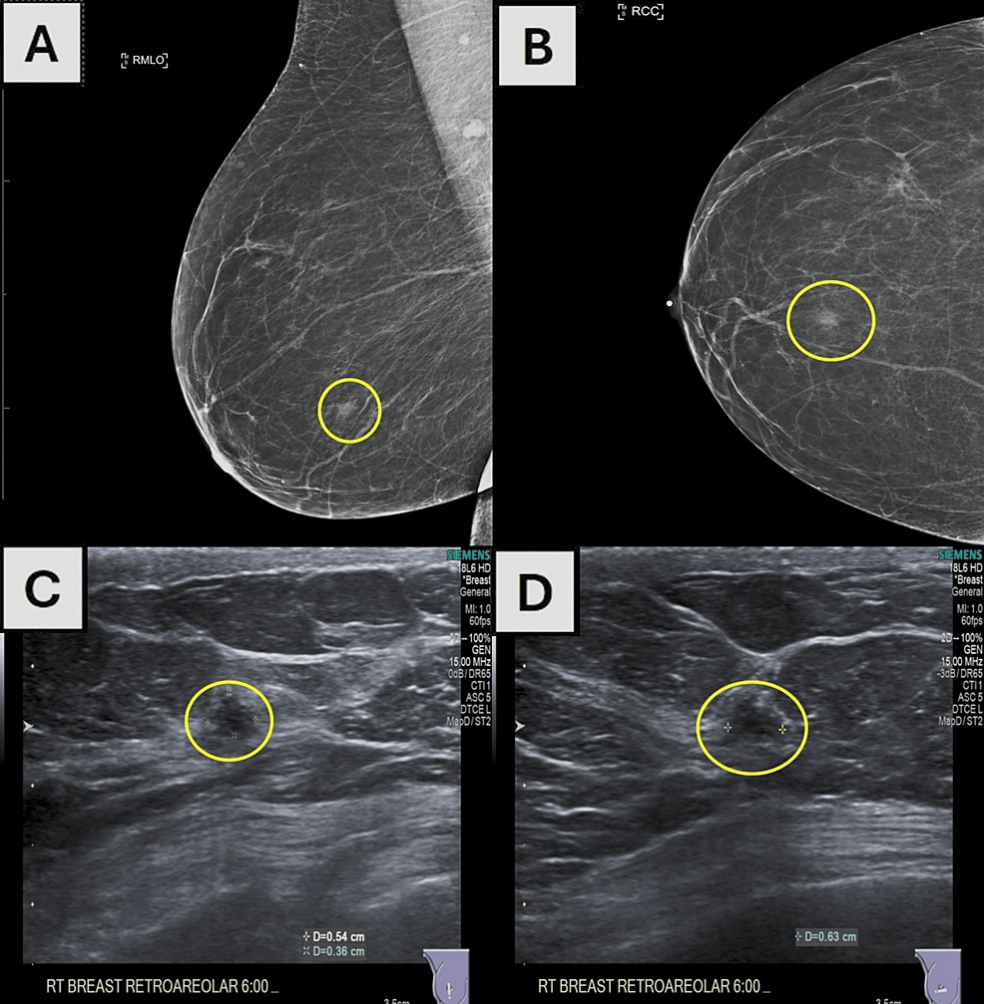
Comprehensive screening and diagnostic imaging of right breast lesion. Initial mammographic images and follow-up ultrasound imaging of the right breast: (A) right mediolateral oblique (RMLO) view and (B) right craniocaudal (RCC) view from screening mammography revealing focal asymmetry in the retroareolar plane, as outlined by the white ovals; (C) ultrasound image depicting a hypoechoic, irregularly shaped mass measuring 0.5 cm × 0.4 cm × 0.6 cm at the retroareolar 6:00 position, as outlined by the white ovals; (D) ultrasound image without internal Doppler color flow corresponding to the area of suspicion. No skin thickening, retraction, or abnormal lymph nodes were observed. The constellation of these findings, not previously noted, was assigned a BI-RADS 5 assessment, suggestive of a high probability of malignancy.

Figure 1 shows a brief graphical representation of the patient's timeline of events. An 80-year-old female initially presented for a routine screening mammogram. She was nulliparous (G0P0), with menarche commencing at 12 years and menopause at 50 years, without any history of hormone replacement therapy. She had a 20-pack-year smoking history but had quit four years prior to the presentation. Her past medical history was significant for coronary artery disease, for which she had undergone stent placements, chronic obstructive pulmonary disease (COPD), hypertension, chronic kidney disease stage III, and she had previously undergone a left total knee replacement. The initial screening mammography, as outlined in Figure 2, revealed a new focal asymmetry in the right breast's retroareolar plane at middle depth, which was not present in previous examinations. This finding was categorized as BI-RADS 0 (incomplete), necessitating additional imaging evaluation. Recommendations included the right diagnostic tomosynthesis and breast ultrasound for a comprehensive assessment.

A diagnostic mammography with tomosynthesis of the right breast was conducted, reaffirming the presence of persistent focal asymmetry within the retroareolar plane in the middle depth and the absence of suspicious calcifications. Concurrent targeted ultrasound, also shown in Figure 2, identified a hypoechoic, irregularly shaped mass measuring 0.5 cm × 0.4 cm × 0.6 cm at the retroareolar 6:00 position, with no internal Doppler color flow, skin thickening, or retraction, and no abnormal lymph nodes in the right axilla. These findings reflected a BI-RADS 5 assessment, indicating high suspicion of malignancy, with a recommendation for an ultrasound-guided needle core biopsy.

**Figure 3:**
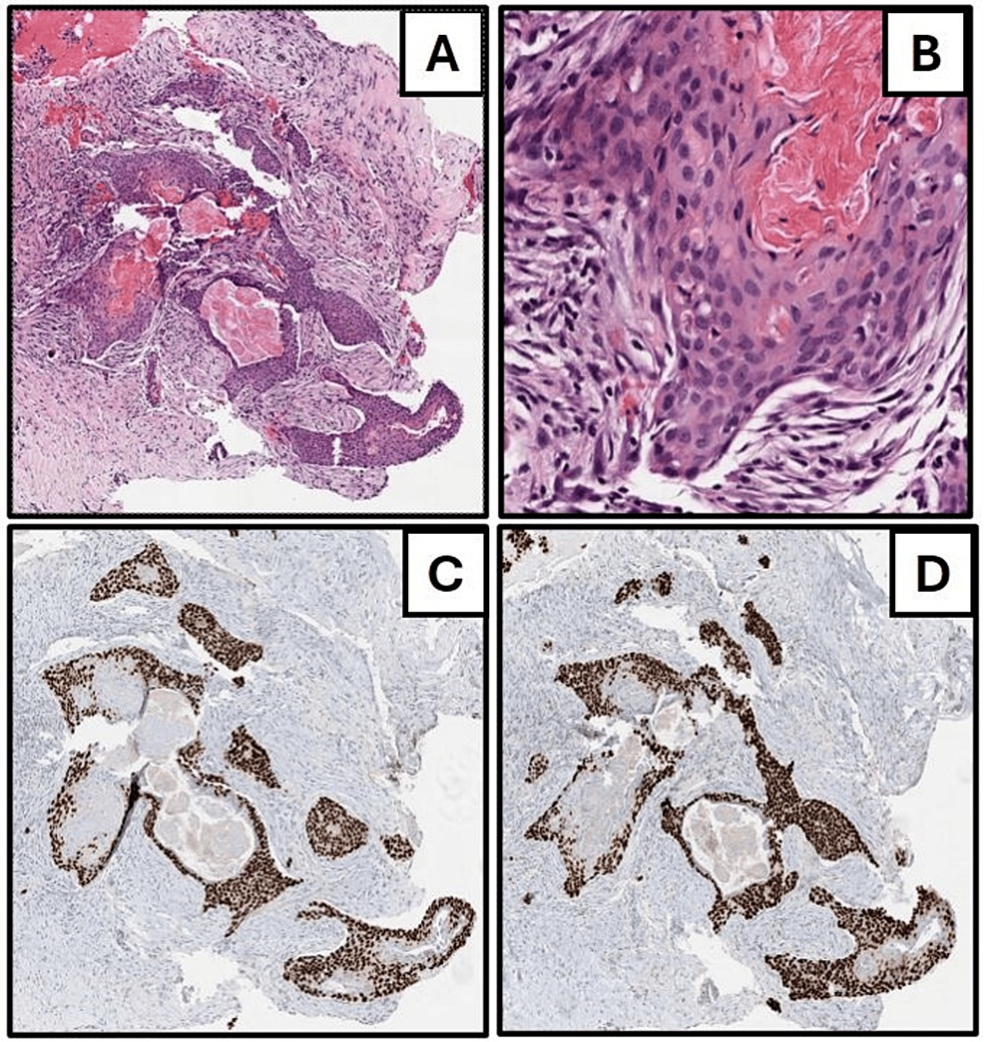
Breast biopsy. This breast biopsy shows nests of keratinizing cysts lined by metaplastic stratified squamous epithelium on hematoxylin and eosin (HE) [(A) upper left, about 40×; (B) upper right, about 200×]. Mild nuclear atypia and pleomorphism are present. The squamous cells are highlighted by diffuse, nuclear positive immunostaining for p40 [(C) lower left, about 40×] and p63 [(D) lower right, about 40×].

The biopsy procedure, performed two weeks later using real-time ultrasound guidance, targeted the aforementioned mass. A total of 10 mL of local anesthetic was administered, and five core samples were obtained. Post-procedure mammograms confirmed the appropriate placement of a coil clip at the biopsy site. Histopathological analysis of the biopsy samples, shown in Figure 3, revealed atypical keratinizing squamous epithelial nests and cysts. Immunohistochemistry (IHC) revealed focal positivity for myoepithelial cells with smooth muscle myosin heavy chain and patchy positivity for p63 and EMA, with negative staining for CEA (monoclonal). The differential diagnosis encompassed a range from ruptured epidermoid cysts with reactions to low-grade adenosquamous carcinoma, among others, recommending an excisional biopsy for a definitive diagnosis.

**Figure 4:**
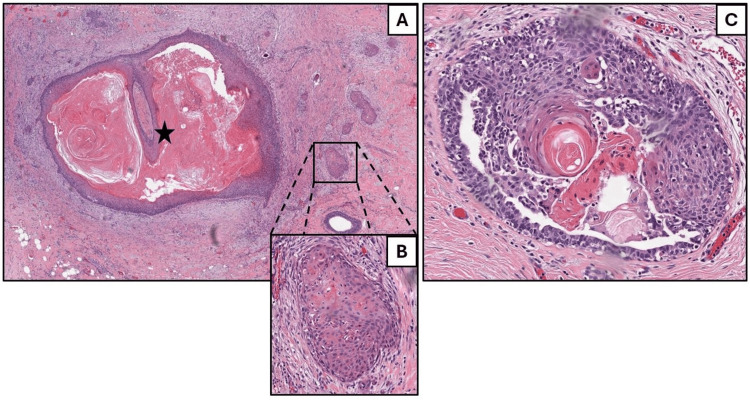
Breast lumpectomy showing primary squamous cell carcinoma of the breast. At low power on hematoxylin and eosin (HE), a similar keratinizing cyst lined by metaplastic stratified squamous epithelium (5-point star) is present (A, left, about 40×). Multiple small nests of squamous tumor cells are infiltrating adjacent stroma (example highlighted in B, about 100×). Near to this tumor focus are foci of breast ducts with squamous metaplasia (C, right, about 200×). No neoplastic spindle cell or glandular elements are identified.

The patient was consulted by a general surgeon shortly after regarding the atypical findings. The plan was set for a lumpectomy to diagnose and treat the retroareolar mass conclusively. Subsequent localization using ultrasound and radiofrequency facilitated the accurate targeting of the mass during the lumpectomy. The lumpectomy specimen underwent comprehensive pathological and IHC analysis, as shown in Figure 4, confirming the diagnosis of an AJCC 8th edition pathological stage IA (pT1b, cN0, cMx), well-differentiated invasive squamous cell carcinoma, measuring 6 mm, situated less than 1 mm from the inferior inked margin. Pertinent negative findings included the absence of ductal carcinoma in situ (DCIS), lymphatic and vascular invasion, dermal lymphovascular invasion, and microcalcifications, as well as no known presurgical therapy effect. A sentinel lymph node examination was not performed, and no regional lymph nodes were submitted or found. All the margins tested negative for invasive carcinoma, but the orientation of the specimen prevented the determination of the inferior margin closest to the invasive carcinoma. IHC staining revealed the estrogen receptor (ER) status as low positive (5%/2+), the progesterone receptor (PR) as negative (0%/0), and the HER2 protein expression as negative (0).

Given the rarity of this entity, the patient’s lumpectomy specimen was sent for expert review at Brigham and Women's Hospital. The consultation detailed the presence of a central keratin-filled cyst with keratinizing stratified squamous epithelium, surrounded by a stroma featuring spindle cells and irregular nests of squamous epithelium, some exhibiting numerous mitoses and extending into adjacent adipose tissue. Despite the unusual histological features suggesting variability in the lesion's origin, including focal squamous metaplasia in adjacent ducts without neoplastic glandular elements, the analysis supported a diagnosis most consistent with a well-differentiated PSCC of the breast. The findings prompted careful consideration of the lesion's characteristics and its proximity to surgical margins, underscoring the importance of excluding extension from an overlying skin lesion through clinical and radiologic evaluations.

Following the pathology report indicating squamous cell carcinoma, the patient had a follow-up with general surgery, leading to a skin examination and the ordering of staging imaging. A thorough skin examination did not reveal any suspicious lesions on the skin of the head, torso, or extremities. A CT of the chest, abdomen, and pelvis showed no suspicious findings, though it noted asymmetric changes in the right breast consistent with recent surgical interventions. A bone scan revealed no evidence of osteoblastic metastases. A bilateral breast MRI revealed a complex picture, notably in the right breast, where a non-mass enhancement exhibited an irregularly shaped distribution within the central aspect across anterior and middle depths, immediately anterior to the excisional biopsy seroma. This area, measuring 1.9 cm in craniocaudal (CC) dimension, 7.2 cm anteroposterior (AP), and 1.9 cm transverse (TR), showed clumped foci of enhancement kinetics suggestive of possible malignancy. Given the spatial relationship between the irregular enhancement and the postoperative seroma, alongside the diagnostic dilemma of differentiating residual disease from postoperative changes, an MRI-guided biopsy was advised to ascertain a definitive diagnosis.

The MRI-guided biopsy involved detailed pre-procedure preparation and execution, concluding with post-biopsy imaging confirming successful sampling and marker clip placement. Pathology from this irregular non-mass enhancement of the right breast found fat necrosis, fibrosis, and a foreign body giant cell reaction, with no evidence of atypical or invasive cells. Following the MRI-guided biopsy, a re-excision with a sentinel lymph node biopsy was conducted due to the initial squamous cell carcinoma diagnosis and unclear margins from the prior biopsy. This procedure revealed no remaining carcinoma within the right breast mass or its inferior margin, with only post-surgical changes and some mild ductal hyperplasia noted. Additionally, the sentinel lymph node biopsy in the right axilla showed no signs of metastatic disease in all three lymph nodes examined (0/3), confirming AJCC 8th edition stage IA (pT1b, pN0(sn), cM0) disease.

Two weeks after surgery, the patient had a follow-up with the general surgeon, showing no postoperative complications, marking the end of this treatment phase. Subsequently, she was evaluated by both radiation and medical oncology for adjuvant treatment considerations. Considering her advanced age, the small size of the lesion, her lack of nodal disease, and her medical comorbidities, the decision was made against adjuvant chemotherapy. This decision was further supported by the limited data on the benefits of such treatment in pT1b PSCC of the breast. Similarly, hormone therapy was not initiated due to the minimal expression of ER and uncertain evidence of its effectiveness in ER-low-positive PSCC. During the radiation oncology evaluation, the patient's physical examination noted well-healed surgical scars and slight nipple discoloration but no other concerns that would contraindicate radiotherapy. After a detailed discussion, a course of hypofractionated right breast radiation therapy was decided upon, delivering a total dose of 42.56 Gy in 16 fractions without an additional boost to the surgical cavity.

**Figure 5:**
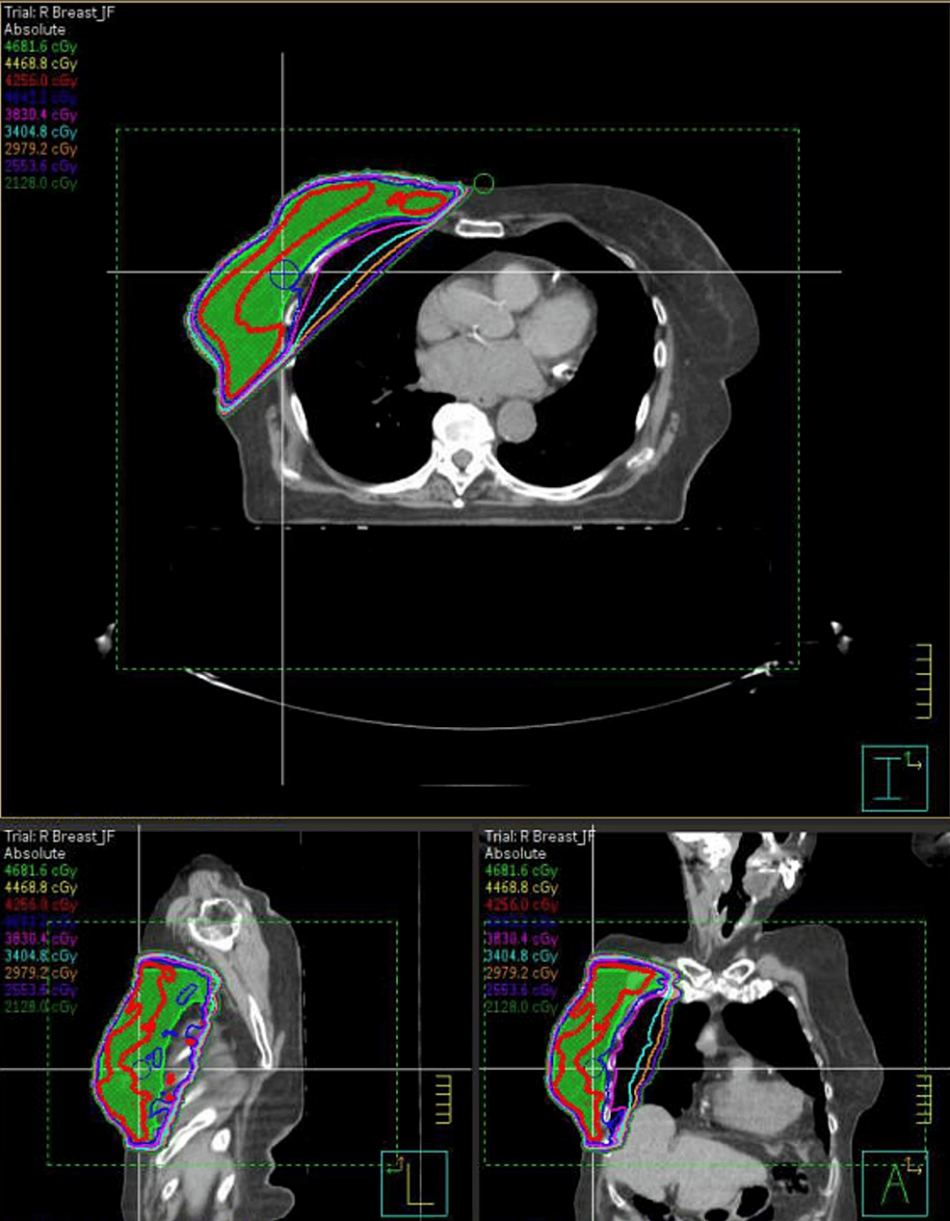
Patient radiation therapy treatment plan. Legend: The isodose lines correlating with the respective colors are displayed in the top left-hand corner of the figure. The planning target volume (PTV) is displayed in a green color.

**Figure 6:**
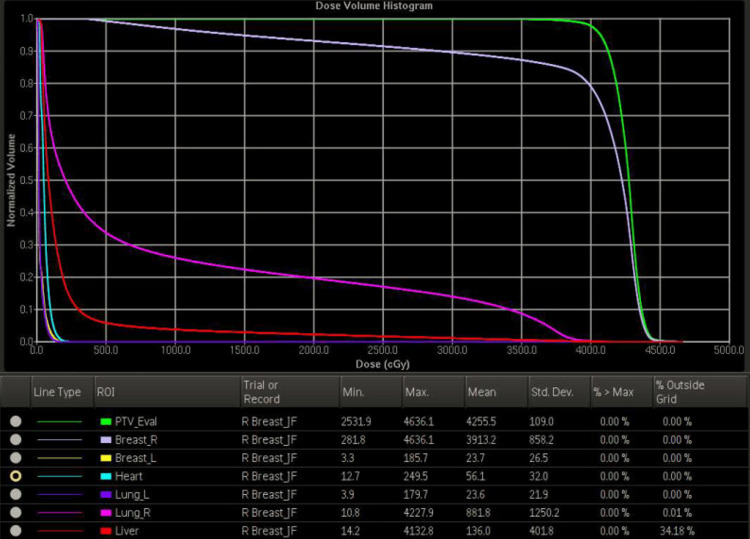
Dose-volume histogram of organs at risk in patient’s radiation treatment plan. Detailed in Figure 5, compiled using Pinnacle Treatment Planning software (Koninklijke Philips N.V., USA).

The treatment plan, as shown in Figures 5-6, employed a 3D conformal technique with mixed 6 and 10 MV photons. At the end of her radiation therapy, the patient exhibited a minimal skin reaction, characterized as acute Common Terminology Criteria for Adverse Events (CTCAE) grade I dermatitis. A follow-up is scheduled in six months, including repeat mammography, and the patient has been advised to maintain regular follow-ups with her other healthcare providers. Three weeks post-radiotherapy, a follow-up appointment with the breast surgeon revealed no abnormalities. Additionally, a nurse-conducted skin check confirmed the resolution of the mild dermatitis experienced by the patient.

## Discussion

PSCC of the breast, first identified by Troell in 1908, remains a rare and enigmatic pathology within the spectrum of breast cancers, with its epidemiology largely undefined due to the scant nature of available data, which primarily consists of database queries and case reports [6]. Yadav et al. conducted a comprehensive Surveillance, Epidemiology, and End Results (SEER) analysis on 445 cases, revealing that the majority of patients were ER-/PR-, less than 20% received a T1 diagnosis, and more than half underwent mastectomy [2]. This analysis also indicated that only a third of the cases received BCT, and a similar proportion were treated with adjuvant radiotherapy [2].

Our contribution to this sparse body of literature is the presentation of an 80-year-old woman diagnosed with an early-stage pT1 PSCC of the breast who was treated with BCT - a clinical decision that is notably rare in this context. Beyond the SEER data, the literature documents only nine instances of adjuvant radiotherapy following PSCC, with merely four of these cases opting for breast conservation therapy, as summarized in Table 1 [7-10]. This case underscores the underexplored demographic and clinical pathways for managing early-stage PSCC with BCT and adjuvant radiotherapy.

**Table 1:** Review of the literature. F: female; MRM: modified radical mastectomy; Gy: gray; SBR: Scarff-Bloom et Richardson; SCC: squamous cell carcinoma; CK: cytokeratin.

First author/pub year	Gender	Age	Breast site	Primary tumor dimensions	ER/PR	Therapy	Prescription	Follow-up	Recurrence	Pathology
Wargotz and Norris [11]	F	53 mean	11 right breasts; 10 left breast; 1 not specified.	39 mm (15–120) mean diameter.	Variable	Mastectomy in 21 patients; 5 patients received adjuvant radiation therapy.	40–50 Gy.	6.2-year median follow up.	8 women developed locoregional recurrence or distant metastases.	19 neoplasms were grade 2 SCC, 2 were grade 1 and 1 was grade 3. Foreign body giant cells in 10 neoplasms. S-100 positive in 5/7 neoplasms tested.
Dejager et al. [12]	F	61	Left breast.	55 mm diameter.	Not provided	Neoadjuvant chemotherapy followed by MRM w/adjuvant radiation therapy.	Not provided.	30 months.	No.	Malignant squamous cells with keratinization.
Rokutanda et al. [9]	F	32	Left breast.	28 mm × 25 mm × 22 mm.	Neg/Neg	Lumpectomy followed by radiation therapy w/adjuvant chemotherapy.	50 Gy in 25 fractions.	12 months.	Yes.	Invasive ductal carcinoma with marked squamous metaplasia.
Gupta et al. [1]	F	43	Right breast.	50 mm × 50 mm × 43 mm.	Neg/Neg	Mastectomy w/adjuvant radiation therapy.	Not provided.	36 months.	No.	Pure primary SCC of the right breast.
Damin et al. [13]	F	39	Right breast.	25 mm × 33 mm × 26 mm.	Neg/Neg	MRM w/adjuvant radiation therapy.	Not provided.	24 months.	No.	Epidermoid carcinoma with extensive areas of necrosis.
Carbone et al. [7]	F	51	Left breast.	25 mm × 18 mm.	Neg/Neg	Lumpectomy w/adjuvant chemotherapy followed by whole breast radiation therapy.	50 Gy in 25 fractions at rate of 2 Gy, with a boost dose of 10 Gy in 5 fractions.	43 months.	No.	Well-differentiated SCC, originated from a focus of squamous metaplasia.
Gupta et al. [14]	F	63	Right and left breast.	30 mm × 60 mm.	Neg/Neg	MRM w/adjuvant chemotherapy followed by radiation therapy.	50 Gy in 25 fractions.	12 months.	No.	Moderately differentiated SCC.
Pribish et al. [8]	F	74	Right breast.	16 mm diameter.	Pos/Neg	Lumpectomy w/adjuvant chemotherapy followed by whole breast radiation therapy.	Not provided.	12 months.	No.	Partly cystic poorly differentiated SCC.
Purkayastha et al. [15]	F	31	Left breast w/axillary involvement.	40 mm × 33 mm.	Neg/Neg	Lumpectomy w/adjuvant chemotherapy followed by whole breast radiation therapy.	50 Gy in 25 fractions.	6 months.	No.	Polyhedral cells with pleomorphic hyperchromatic nuclei, prominent nucleoli and individual cell keratinization.
Shrestha et al. [10]	F	18	Left breast.	100 mm diameter.	Neg/Neg	Lumpectomy w/adjuvant chemotherapy followed by whole breast radiation therapy.	Not provided.	9 months.	No.	Poorly differentiated SCC positive for CK, CK7, p40 with a Ki67 proliferation index of 70%.
Qasseh et al. [16]	F	42	Left breast.	60 mm diameter.	Neg/Neg	MRM w/adjuvant chemotherapy then adjuvant radiation therapy.	40 Gy.	30 months.	Yes.	SBR grade III invasive squamous cell metaplastic breast.

A crucial step in confirming a PSCC diagnosis is to methodically exclude any potential for extramammary SCC metastasis, typically originating from sites such as the skin, lung, esophagus, cervix, or urinary bladder [17]. Our patient underwent comprehensive staging imaging that revealed no signs of metastasis, reinforcing the likelihood of primary breast pathology. Subsequently, accurate diagnosis requires careful examination of the pathological features synonymous with PSCC, as defined by the World Health Organization (WHO): a metaplastic carcinoma that often presents as a cystic mass lined with atypical or pleomorphic metaplastic squamous cells and variable keratinization, which is consistent with the findings exhibited in Figures 2-3 [18]. Nests, sheets, or cords of infiltrative tumor cells often invade adjacent stroma, eliciting a myxoid, desmoplastic stromal reaction, and inflammation, and squamous metaplasia may be detected in nearby ducts (as shown in Figure 4). In accordance with Rosen’s Breast Pathology, pure SCC should be comprised of at least 90% of squamous cells in the tumor cell population, a criterion that excludes a mixed-type carcinoma (IBC-NST; i.e., adenosquamous) with significant spindle cell or glandular components, which our case did not exhibit [19]. The presence of this squamous population and the lack of neoplastic spindle cells or glandular features steered the diagnosis away from a mixed metaplastic carcinoma. The IHC profile of our case, which showed positivity for p40 and p63, helps solidify the diagnosis, with p63 having an approximate sensitivity and specificity of 86.7% and 99.4%, respectively [20,21]. Our patient's clinical and histopathological profile satisfies these criteria, affirming the diagnosis of PSCC.

Given the patient's minimal ER positivity and the ambiguous benefits of endocrine therapy in such instances, the patient’s disease was treated as early-stage triple-negative breast cancer (TNBC), reflecting a tailored approach to management based on the comprehensive assessment of pathological findings and clinical context [22]. The treatment landscape for PSCC remains largely undefined, with no consensus on the optimal therapeutic approach. While surgery forms the cornerstone of treatment, the role of neoadjuvant and adjuvant therapies, particularly chemotherapy and radiotherapy, is less clear. In this report, we treated the patient at an early stage (T1b, TNBC). Generally, the management of early-stage, node-negative TNBC involves the administration of neoadjuvant chemotherapy, now increasingly combined with immunotherapy as recommended by the findings of the KEYNOTE 522 and IMpassion031 trials [23-25]. This is followed by a surgical intervention. For cases presenting with residual disease post-surgery, adjuvant chemotherapy is considered, drawing on evidence from the CREATE-X trial [26]. Subsequently, adjuvant radiation therapy is considered a potential component of the comprehensive treatment strategy. Given her advanced age and comorbidities, our decision to omit chemotherapy and immunotherapy in favor of radiotherapy was influenced by the tumor's T1b classification, drawing parallels to outcomes observed in T1a and T1b TNBC cases, which exhibit excellent prognoses without adjuvant chemotherapy [27].

## Conclusions

Our report adds to the limited pool of data on adjuvant radiation therapy following lumpectomy for early-stage PSCC, marking it as one of the few documented case reports of BCT. The paucity of evidence underscores the necessity of individualized treatment plans and highlights the need for further research to establish evidence-based guidelines for managing this rare disease entity. The successful outcome of our patient's treatment, characterized by the omission of chemotherapy and the implementation of adjuvant radiotherapy, offers valuable insights into the potential therapeutic pathways for similar cases in the future.
